# Supplementary material for: Y Chromosome, Mitochondrial DNA and Childhood Behavioural Traits
Source: Sci Rep. 2017 Sep 14;7:11655. doi: 10.1038/s41598-017-10871-4 (PMC5599552; doi:10.1038/s41598-017-10871-4)
Supplement: Supplementary file 1 — Supplementary Materials [file 41598_2017_10871_MOESM1_ESM.pdf]

Y chromosome, mitochondrial DNA and childhood behavioural traits

Laurence J. Howe, MSc, A. Mesut Erzurumluoglu, PhD, George Davey Smith, MD, DSc, Santiago Rodriguez, PhD, Evie Stergiakouli, PhD*.

Correspondence: Dr. Evie Stergiakouli, MRC Integrative Epidemiology Unit (IEU) at the University of Bristol, University of Bristol, Barley House, Oakfield Grove, BS8 2BN, Bristol, UK; e-mail: e.stergiakouli@bristol.ac.uk.

Supplementary Material

Supplementary Methods. ALSPAC genotyping & mitochondrial DNA haplogroup derivation

Table S1. Pairwise tetrachoric correlations between binary behavioural trait variables

Table S2. Association of major Y chromosome haplogroups in ALSPAC with the number of behavioural traits adjusted for paternal social class and GCSE results

Table S3: Association of major mitochondrial DNA haplogroups in ALSPAC with the number of behavioural traits adjusted for maternal and paternal social class and GCSE results

Table S4. Odds ratios of Y chromosome haplogroups subgroups on binary psychiatric trait measures from logistic regression adjusted for paternal social class and GCSE results

Table S5. Association of Y chromosome haplogroup subgroups in ALSPAC with the number of behavioural traits adjusted for paternal social class and GCSE results

Table S6. Odds ratios of mitochondrial DNA chromosome haplogroup subgroups on binary behavioural trait measures from logistic regression adjusted for paternal social class and GCSE results

Table S7. Association of mitochondrial DNA haplogroup subgroups in ALSPAC with the number of behavioural traits adjusted for maternal and paternal social classes and GCSE results

Supplementary Figure 1. Y chromosome and Mitochondrial DNA haplogroups in ALSPAC compared to independent English populations

Supplementary Methods

ALSPAC genotyping quality control

Individuals were excluded from further analysis on the basis of having incorrect gender assignments, minimal or excessive heterozygosity (< 0.320 and > 0.345 for the Sanger data and < 0.310 and > 0.330 for the LabCorp data), disproportionate levels of individual missingness (> 3%), evidence of cryptic relatedness (> 10% IBD) and being of non-European ancestry (as detected by a multidimensional scaling analysis seeded with HapMap 2 individuals). EIGENSTRAT analysis revealed no additional obvious population stratification and genome-wide analyses with other phenotypes indicate a low lambda) (23). SNPs with a minor allele frequency of < 1% and call rate of < 95% were removed. After QC, 8,365 unrelated individuals were available for analysis.

Mitochondrial DNA haplogroup derivation

7,554 custom mitochondrial probes, targeting 2,824 unique mtDNA positions, were included on the Illumina HumanHap550 quad genome-wide SNP genotyping platform. All heterozygous genotype calls (i.e. heteroplasmy) were set to missing prior to quality control using PLINK (Purcell, Neale *et al*. 2007). Genotype calls obtained from each probe were compared to the human mitochondrial database of non-pathological mitochondrial sequence variants (www.hmtdb.uniba.it:8080/hmdb/) to ensure that known allelic variants were being called. Probes were excluded in cases where genotype calls were not represented in the Cambridge Reference Sequence reference (rCRS) or one of the known allelic variants. Probes with an overall call rate of < 95% were excluded prior to analysis. The genotyping concordance of the remaining probes was investigated by comparing the genotype calls in 445 replicate samples. With the exception of probe failure (i.e. missing data), a 100% genotyping concordance rate was obtained for each probe. All probes with a failure rate of >

5% in the replicate sample were further excluded. In cases where multiple probes passed the above mentioned QC criteria, the probe with the highest calling rate was used for analysis. A total of 1062 probes passed QC for the batch that was genotyped by Laboratory Corporation of America (n=7590), whilst 629 probes passed QC for the batch genotyped by the Sanger Institute (n=775). Haplogroup assignment was performed as described by Kloss-Brandstatter et al (25), and samples with a quality score of more than 90% were used for our analysis. Major haplogroups were defined as containing multiple haplogroups that are closely related to utilize information on less common haplogroups. After QC and removing individuals with withdrawn consent; our data-set contained 4,211 males and 4,009 females with derived mitochondrial DNA haplogroups.

Table S1. Pairwise tetrachoric correlations between binary behavioural trait variables

	AASS	ABS	TBS	SCDC	Hyper	Conduct	Emotional	Total	PLIKS 14	PLIKS 18
**AASS**	1	-	-	-	-	-	-	-	-	-
**ABS**	0.57	1	-	-	-	-	-	-	-	-
**TBS**	0.39	0.33	1	-	-	-	-	-	-	-
**SCDC**	0.67	0.67	0.48	1	-	-	-	-	-	-
**Hyper**	0.72	0.37	0.33	0.48	1	-	-	-	-	-
**Conduct**	0.48	0.51	0.45	0.58	0.47	1	-	-	-	-
**Emotional**	0.20	0.22	0.09	0.28	0.17	0.28	1	-	-	-
**Total**	0.67	0.48	0.40	0.63	0.72	0.72	0.67	1	-	-
**PLIKSi 14**	0.14	0.11	0.08	0.05	0.10	0.09	0.11	0.11	1	-
**PLIKSi 18**	0.07	0.11	0.04	0.04	0.11	0.09	0.13	0.14	0.46	1

AASS: Attention/activity symptoms score; ABS: Awkward behaviours score; TBS: Troublesome behaviours score; SCDC: Social and communication disorder checklist; PLIKSi: Psychosis like symptoms

Table S2. Association of major Y chromosome haplogroups in ALSPAC with the number of behavioural traits adjusted for paternal social class and GCSE results

	Effect size of major Y chr haplogroup on behavioural traits: Beta (95% C.I.) R haplogroup is the reference		Adjusted P values
Behavioural/Psychiatric trait score	I	Other	
Attention/ Activity symptoms score (DAWBA)	0.16 (-0.51, 0.83)	1.36 (0.19, 2.53)	0.075
Awkward behaviours score (DAWBA)	-0.03 (-0.30, 0.25)	0.18 (-0.31, 0.66)	0.81
Troublesome behaviours score (DAWBA)	0.00 (-0.10, 0.10)	0.01 (-0.17 , 0.18)	1.00
SCDC	0.06 (-0.31, 0.44)	0.25 (-0.41, 0.90)	0.74
Hyperactivity traits (SDQ)	0.06 (-0.17, 0.28)	0.35 (-0.04, 0.74)	0.20
Conduct traits (SDQ)	0.17 (0.03, 0.30)	0.02 (-0.22, 0.26)	0.06
Emotional symptoms (SDQ)	0.04 (-0.11, 0.20)	0.19 (-0.09, 0.46)	0.38
Total behavioural traits (SDQ)	0.12 (-0.33, 0.56)	0.65 (-0.13, 1.43)	0.25
PLIKSi age 14	-0.01 (-0.05, 0.02)	-0.01 (-0.07, 0.06)	0.76
PLIKSi age 18	0.00 (-0.04, 0.03)	0.01 (-0.05, 0.08)	0.92

95% CI: 95% Confidence Interval; DAWBA: Development and Well-Being Assessment; SCDC: Social and communication disorders checklist; SDQ: Strengths and Difficulties Questionnaire; PLIKSi: Psychosis-Like Symptom Interview

Table S3: Association of major mitochondrial DNA haplogroups in ALSPAC with the number of behavioural traits adjusted for maternal and paternal social class and GCSE results

	Odds ratios of major mitochondrial DNA haplogroup on behavioural and psychiatric traits: OR (95% C.I.) HV haplogroup is the reference					Adjusted P values
**Behavioural/ Psychiatric trait score**	**J**	**K**	**TR**	**U**	**Other**	
**Attention/ Activity symptoms score (DAWBA)**	-0.35 (-1.01, 0.32)	-0.30 (-1.04, 0.44)	0.03 (-0.62, 0.69)	0.20 (-0.43, 0.83)	-0.14 (-0.91, 0.64)	0.78
**Awkward behaviours score (DAWBA)**	-0.13 (-0.41, 0.16)	-0.06 (-0.38, 0.25)	-0.06 (-0.34, 0.22)	0.06 (-0.21, 0.32)	-0.08 (-0.41, 0.25)	0.91
**Troublesome behaviours score (DAWBA)**	-0.03 (-0.14, 0.07)	-0.02 (-0.13, 0.10)	0.01 (-0.09, 0.11)	0.05 (-0.05, 0.15)	0.06 (-0.06, 0.18)	0.74
**SCDC**	-0.26 (-0.63, 0.12)	-0.25 (-0.66, 0.17)	-0.20 (-0.57, 0.17)	0.05 (-0.30, 0.40)	-0.22 (-0.66, 0.22)	0.50
**Hyperactivity traits (SDQ)**	-0.03 (-0.26, 0.20)	0.05 (-0.20, 0.31)	0.21 (-0.02, 0.45)	0.05 (-0.17, 0.26)	0.06 (-0.21, 0.33)	0.59
**Conduct traits (SDQ)**	0.08 (-0.06, 0.23)	-0.07 (-0.23, 0.09)	0.09 (-0.06, 0.23)	0.03 (-0.11, 0.16)	-0.02 (-0.20, 0.15)	0.58
**Emotional symptoms (SDQ)**	0.08 (-0.09, 0.26)	-0.10 (-0.29, 0.09)	-0.07 (-0.24, 0.11)	0.03 (-0.13, 0.20)	-0.16 (-0.36, 0.05)	0.35
**Total behavioural traits (SDQ)**	0.18 (-0.30, 0.65)	-0.03 (-0.55, 0.48)	0.23 (-0.24, 0.70)	0.14 (-0.30, 0.58)	-0.26 (-0.81, 0.29)	0.70
**PLIKSi age 14**	0.03 (-0.08, 0.14)	-0.07 (-0.19, 0.04	0.04 (-0.06, 0.15)	-0.07 (-0.17, 0.03)	-0.02 (-0.15, 0.10)	0.37
**PLIKSi age 18**	-0.02 (-0.15, 0.11)	0.02 (-0.11, 0.16)	0.07 (-0.05, 0.20)	-0.02 (-0.13, 0.10)	-0.14 (-0.29, 0.01)	0.29

95% CI: 95% Confidence Interval; DAWBA: Development and Well-Being Assessment; SCDC: Social and communication disorders checklist; SDQ: Strengths and Difficulties Questionnaire; PLIKSi: Psychosis-Like Symptom Interview

Table S4. Odds ratios of Y chromosome haplogroups subgroups on binary psychiatric trait measures from logistic regression adjusted for paternal social class and GCSE results

Behavioural/ Psychiatric trait score	P Values			
	N	Unadjusted	N	Adjusted
**Attention/ Activity symptoms score (DAWBA)**	3265	0.26	2400	0.25
**Awkward behaviours score (DAWBA)**	3251	0.67	2390	0.48
**Troublesome behaviours score (DAWBA)**	3262	0.29	2396	0.58
**SCDC**	3253	0.39	2392	0.16
**Hyperactivity traits (SDQ)**	3300	0.64	2461	0.63
**Conduct traits (SDQ)**	3304	0.76	2460	0.43
**Emotional symptoms (SDQ)**	3301	0.06	2460	0.09> 0.006[1]
**Total behavioural traits (SDQ)**	3298	0.74	2457	0.78
**PLIKSi age 14**	2859	0.95	2059	0.98
**PLIKSi age 18**	1749	0.74	1228	0.56

95% CI: 95% Confidence Interval; DAWBA: Development and Well-Being Assessment; SCDC: Social and communication disorders checklist; SDQ: Strengths and Difficulties Questionnaire; PLIKSi: Psychosis-Like Symptom Interview

1 Smallest adjusted p value compared to multiple testing threshold

Table S5. Association of Y chromosome haplogroup subgroups in ALSPAC with the number of behavioural traits adjusted for paternal social class and GCSE results

Behavioural/ Psychiatric trait score	P Values			
	**N**	**Unadjusted**	**N**	**Adjusted**
**Attention/ Activity symptoms score (DAWBA)**	3265	0.40	2400	0.07
**Awkward behaviours score (DAWBA)**	3251	0.71	2390	0.22
**Troublesome behaviours score (DAWBA)**	3262	0.78	2396	0.59
**SCDC**	3253	0.39	2397	0.05>0.006[2]
**Hyperactivity traits (SDQ)**	3300	0.64	2461	0.63
**Conduct traits (SDQ)**	3304	0.61	2462	0.26
**Emotional symptoms (SDQ)**	3301	0.14	2462	0.20
**Total behavioural traits (SDQ)**	3298	0.76	2459	0.29
**PLIKSi age 14**	2859	0.68	2062	0.53
**PLIKSi age 18**	1749	0.11	1270	0.13

95% CI: 95% Confidence Interval; DAWBA: Development and Well-Being Assessment; SCDC: Social and communication disorders checklist; SDQ: Strengths and Difficulties Questionnaire; PLIKSi: Psychosis-Like Symptom Interview

1 Smallest adjusted p value compared to multiple testing threshold

Table S6. Odds ratios of mitochondrial DNA chromosome haplogroup subgroups on binary behavioural trait measures from logistic regression adjusted for paternal social class and GCSE results

Behavioural/ Psychiatric trait score	P Values			
	N	Unadjusted	N	Adjusted
**Attention/ Activity symptoms score (DAWBA)**	5674	0.95	3857	0.88
**Awkward behaviours score (DAWBA)**	5646	0.20	3841	0.66
**Troublesome behaviours score (DAWBA)**	5649	0.90	3843	0.83
**SCDC**	5651	0.19	3850	0.21
**Hyperactivity traits (SDQ)**	5731	0.71	3926	0.90
**Conduct traits (SDQ)**	5738	0.90	3925	0.58
**Emotional symptoms (SDQ)**	5731	0.23	3927	0.12> 0.006[1]
**Total behavioural traits (SDQ)**	5723	0.77	3922	0.35
**PLIKSi age 14**	5154	0.44	3426	0.42
**PLIKSi age 18**	3516	0.18	2369	0.63

95% CI: 95% Confidence Interval; DAWBA: Development and Well-Being Assessment; SCDC: Social and communication disorders checklist; SDQ: Strengths and Difficulties Questionnaire; PLIKSi: Psychosis-Like Symptom Interview

1 Smallest adjusted p value compared to multiple testing threshold

Table S7. Association of mitochondrial DNA haplogroup subgroups in ALSPAC with the number of behavioural traits adjusted for maternal and paternal social classes and GCSE results

Behavioural/ Psychiatric trait score	P Values			
	N	Unadjusted	N	Adjusted
**Attention/ Activity symptoms score (DAWBA)**	5674	0.65	3847	0.99
**Awkward behaviours score (DAWBA)**	5646	0.28	3841	0.53
**Troublesome behaviours score (DAWBA)**	5649	0.85	3843	0.98
**SCDC**	5651	0.11	3850	0.16
**Hyperactivity traits (SDQ)**	5731	0.71	3926	0.90
**Conduct traits (SDQ)**	5738	1.00	3929	0.74
**Emotional symptoms (SDQ)**	5731	0.18	3927	0.25
**Total behavioural traits (SDQ)**	5723	0.95	3922	0.90
**PLIKSi age 14**	5154	0.32	3429	0.23
**PLIKSi age 18**	3516	0.03	2370	0.01> 0.006[1]

95% CI: 95% Confidence Interval; DAWBA: Development and Well-Being Assessment; SCDC: Social and communication disorders checklist; SDQ: Strengths and Difficulties Questionnaire; PLIKSi: Psychosis-Like Symptom Interview

1 Smallest adjusted p value compared to multiple testing threshold

Supplementary Figure 1: Y chromosome and Mitochondrial DNA haplogroups in ALSPAC compared to independent English populations

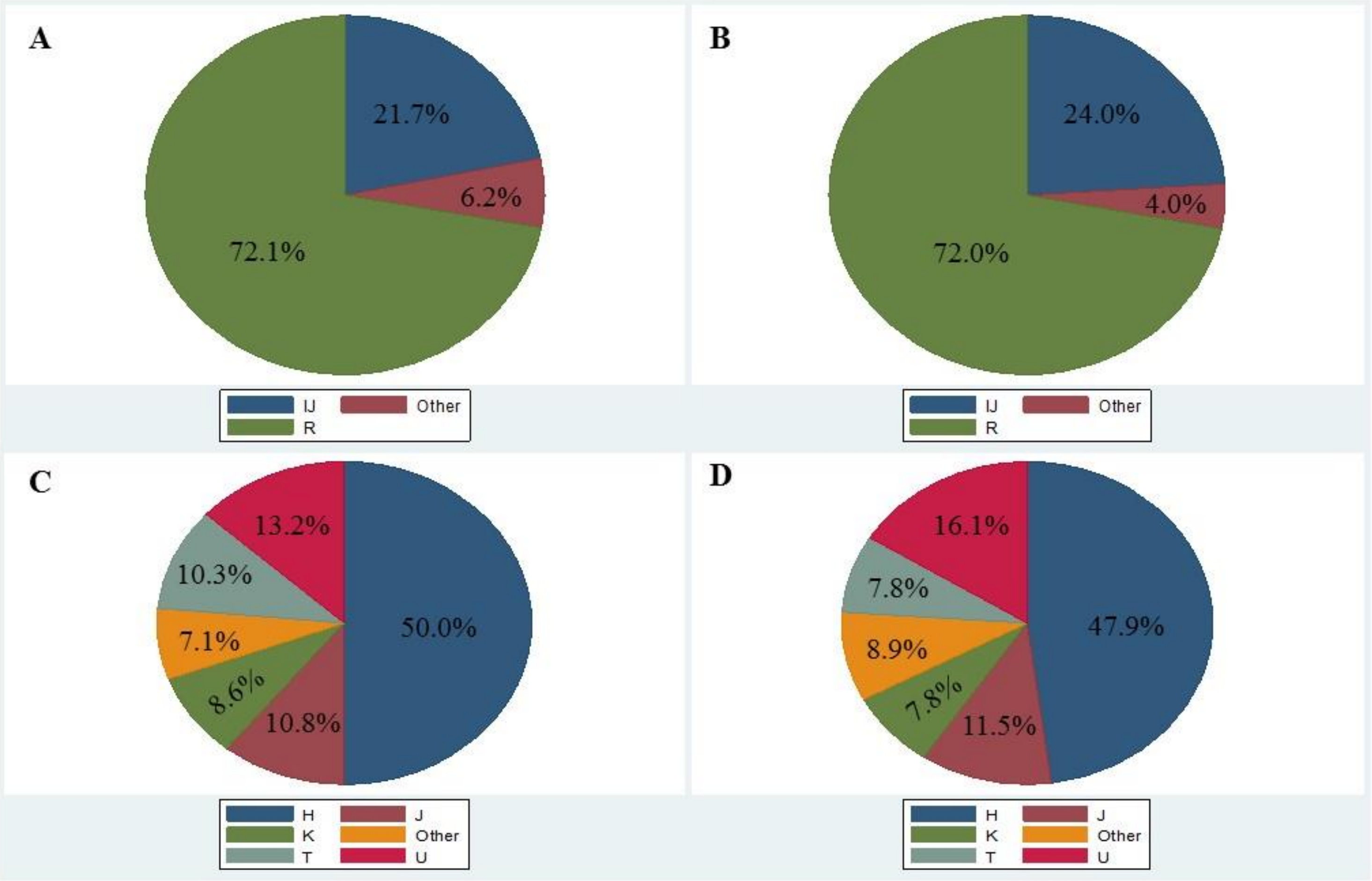

A: Y chromosome haplogroups in ALSPAC (N = 4,788)

B: Y chromosome haplogroups in England (independent) via Eupedia (N > 250)

C: Mitochondrial DNA haplogroups in ALSPAC (N = 8,220)

D: Mitochondrial DNA haplogroups in England (independent) via Eupedia (N = 2,333)
